# A Case of Symptomatic Cerebral Radiation Necrosis for an Extra-Cranial Neoplasm from Conventional Radiotherapy With Concurrent Immunotherapy

**DOI:** 10.7759/cureus.15712

**Published:** 2021-06-17

**Authors:** Radhika Gutta, Nino Balanchivadze, Ding Wang

**Affiliations:** 1 Internal Medicine, Henry Ford Health System, Detroit, USA; 2 Hematology and Oncology, Henry Ford Health System, Detroit, USA

**Keywords:** radiation necrosis, immunotherapy, radiotherapy, cancer, immune checkpoint therapy

## Abstract

Cerebral radiation necrosis (RN) is a known complication of brain radiotherapy (RT). The incidence rate of RN varies with the total dose, dose fractionation, and radiotherapy modality. Concurrent treatment with immunotherapy can increase the risk factors for developing RN through a synergistic mechanism. Here, we describe a patient who developed cerebral RN after receiving conventional RT to an extra-cranial site, while he was receiving immune checkpoint inhibitor (ICI) therapy.

## Introduction

Cerebral radiation necrosis (RN) is an expected complication of brain radiotherapy (RT), especially with the high dose and fraction used in stereotactic radiosurgery. It typically occurs 3 to 12 months following brain RT. However, delayed cerebral RN has been observed even several years after brain RT [1]. Various risk factors can contribute to RN development, yet it has rarely occurred at brain RT doses within 50 Gy into 25 fractions [2]. Here, we describe a patient who developed cerebral RN after receiving a modest RT level of 30 Gy in 5 fractions to the right cervical lymphatic drainage, which occurred while he was receiving immune checkpoint inhibitor (ICI) therapy. This patient later developed neurologic symptoms and showed radiological evidence of cerebral RN. The extra-cranial RT field, as well as the conventional RT dose, led to delays in his diagnosis with initial concerns directed towards metastatic disease. With this case, we highlight that cerebral RN can occur with low dose RT at extra-cranial sites, especially in patients receiving concurrent immunotherapy, and that physicians should be attentive to patient characteristics that may increase the risk of developing cerebral RN.

## Case presentation

**Figure 1:**
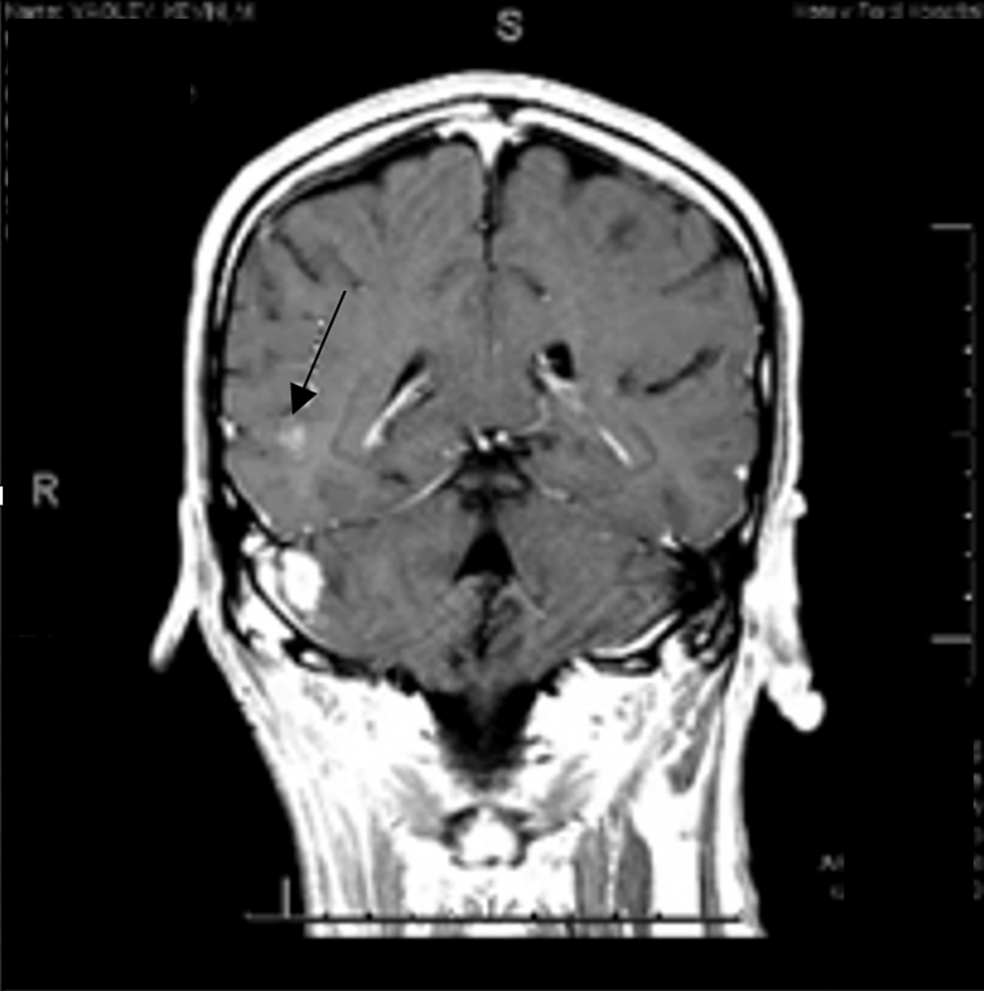
MRI with initial concerns of metastasis vs. radiation induced changes There are new and increasing areas of restricted diffusion within the subcortical white matter of the right temporal lobe. These findings were initially described as nonspecific and differential considerations included cerebellitis/cerebritis, radiation injury, and metastatic disease.

A 40-year-old man with no significant medical history presented to the oncology clinic with a new diagnosis of stage IIIC malignant melanoma that involved the right parotid gland with extension into the right cervical lymphatic chain. His initial staging workup, including brain magnetic resonance imaging (MRI) and computed tomography (CT) scan of the chest, abdomen and pelvis ruled out distant metastasis. Right parotid lymph node biopsy was performed and confirmed malignant melanoma with BRAF positive status. Systemic treatment with ipilimumab and nivolumab was initiated, with subsequent total parotidectomy and comprehensive neck dissection and flap reconstruction. During his first cycles of combination therapy with ipilimumab and nivolumab, he also received postoperative RT at 30 GY in 5 fractions delivered over 2 weeks. The RT field was targeted at the right intra-parotid lymph node that measured around 3 cm in size. After 2 cycles of combination immunotherapy, the patient presented to the emergency room (ER) with high grade fevers. He underwent infectious workup including blood cultures, urinalysis, and CT of the chest, abdomen, pelvis. His fevers were ultimately attributed to immunotherapy and he was started on oral prednisone 100 mg daily with a taper. The ipilimumab was discontinued after the second cycle in the setting of these fevers. The patient then received 17 cycles of nivolumab monotherapy until he presented to the emergency department with headaches and generalized seizures. His brain MRI revealed increased T2 flair signal abnormality within the right lateral cerebellum and new areas of restricted diffusion within the subcortical white matter of the right temporal lobe, with a contrast-enhanced ring-enhancing lesion (Figure 1).

**Figure 2:**
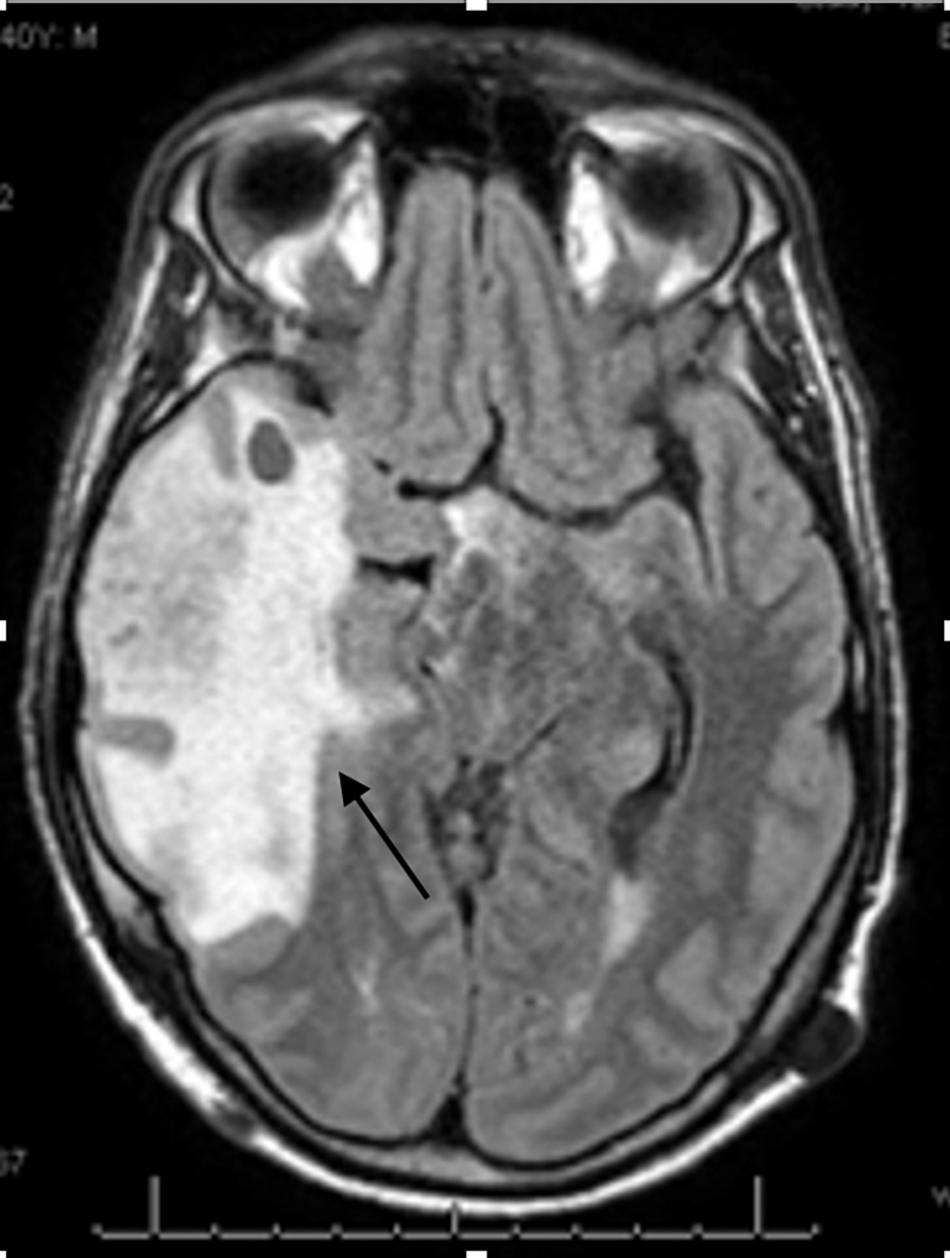
Progression of radiation necrosis Interval increase in size of the enhancing lesions within the right temporal lobe with related increase in surrounding T2/FLAIR hyperintense signal and increase in mass effect upon the right lateral ventricle and midline shift to the left.

His presentation was initially concerning for brain metastasis instead of radiation injury, even though the patient had no evidence of extra-cranial systemic disease recurrence. The patient had persistent symptoms for which he was treated with dexamethasone 1 mg three times a day, including headaches and seizures requiring anti-epileptics. His follow-up brain MRI showed a persistent contrast-enhancing right lateral temporal lesion with surrounding worsening vasogenic edema (Figure 2).

**Figure 3:**
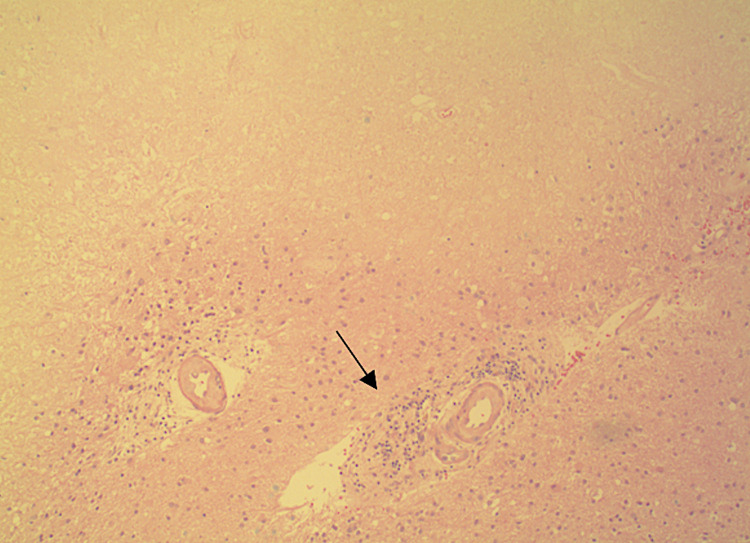
Pathology showing adjacent inflammation and gliosis with extensive multifocal radiation necrosis. HMB-45 and Melan A immunostains done were negative, which indicates no evidence of tumor.

The patient went through craniotomy and resection of the contrast-enhancing right temporal lesion for decompression and tissue diagnosis. Pathology observed no evidence of metastatic melanoma. However, extensive multifocal RN surrounded by adjacent inflammation and gliosis was observed (Figure 3). HMB-45 and Melan A immunostains, which are commonly indicative of melanoma, were negative on the brain biopsy tissue. The patient recovered well postoperatively and remained off of dexamethasone without recurrent symptoms of headache, nausea, and vomiting. He has remained off of systemic ICI therapy over the last 2 years and has resumed full-time work with no evidence of melanoma brain metastasis or systemic recurrence.

## Discussion

Cerebral RN is a known complication of brain RT but typically is not expected from extra-cranial RT. The incidence of RT-induced cerebral RN, in general, varies based on RT treatment parameters such as dose fractionation, modality of administration, total dose, the targeted RT field, and whether RT has been administered with concurrent chemotherapy or immunotherapy. Cerebral RN rarely develops from whole brain RT of less than 50 Gy [2].

The histopathology of RN includes liquefication necrosis and coagulation in the white matter with capillary breakdown and hyalinization of blood vessels [3]. Hypoxia of the surrounding tissues that accompanies RT activates the up upregulation of hypoxia-inducible factor-1 alpha (HIF-1α) in human glucose transporter 5 (hGLUT5) and CD68-positive microglia [3]. As a consequence, VEGF pathway signaling is activated leading to leaky angiogenesis and the development of edema around the lesion [4].

Apart from direct tissue damage expected with the use of ionizing radiotherapy, other mechanisms, such as immune activation likely contributes to the development of RN. The interaction of RT with the immune system is complex [5,6]. Ionizing radiation is thought to increase the immunogenicity of tumor cells by inducing immunogenic cell death and can affect the immunosuppressive tumor environment. In addition, RT promotes the secretion of pro-inflammatory cytokines that operate at the local level to result in cell death of nearby cells [7]. The response is not only locally, as there can be a systemic response to non-therapeutic doses of RT, referred to as the abscopal effect, that has been observed in patients with malignant melanoma treated with immunotherapy [8]. This highlights the complex process of immune activation that could potentially explain potentiating radiation injury even with non-cranial intended RT. Overlapping immunotherapy with RT has been implicated as a factor for an increased risk of developing RN [9]. Ipilimumab, a monoclonal antibody that is cytotoxic to T-lymphocyte antigen (CTLA-4), penetrates the blood-brain barrier at leaky sites and activates cytotoxic T-lymphocytes to infiltrate the tumor site and induce anti-tumor effects [10], and it has been shown to improve overall survival in patients with malignant melanoma [11]. Reported cases have shown an increased incidence of RN in patients receiving concurrent ipilimumab therapy and RT [10]. One recent study showed that patients with melanoma brain metastasis who were treated with stereotactic radiosurgery and ipilimumab had a higher incidence of RN than patients who did not receive ipilimumab (6%-8% vs 0%; P=0.005) [12]. Also, a retrospective study that assessed 180 patients showed that patients who received immunotherapy had higher rates of RN than those who received only stereotactic radiosurgery (37.5% vs 4.7%) [9]. 

Patients with RN can be asymptomatic or may have symptoms reflecting increased intracranial pressure due to surrounding edema, such as headaches, cognitive changes, focal neurological deficits, and seizures. The signs can be subtle and often do not correlate with the degree of imaging abnormalities [1].

The diagnosis of RN is complex, as it can mimic other pathologies on imaging. On CT imaging and T1 and T2 weighted MRI, RN may look like a residual or new tumor. Tumors and RN share several imaging characteristics, such as contrast enhancement with surrounding edema, and both tumors and RN can increase over time [13]. Currently, a biopsy of the lesion is the only definite method for the diagnosis of RN [1].

Corticosteroids are indicated as first-line treatment for RN [3]. Studies have also shown some benefits of using bevacizumab, an anti-VEGF monoclonal antibody, due to its potential to lessen the edema associated with necrosis and improve neurological symptoms [14]. In cases where therapies are unsuccessful at alleviating RN, or if patients show increasing intracranial pressure, surgical resection of the site of necrosis may be needed [15].

The differential diagnosis for RN should be high for patients who are receiving immunotherapy in conjunction with RT, and ignoring this possibility could lead to misdiagnosis and incorrect treatment. Because of our patient’s experience and the compelling evidence of possible adverse interactions between RT and ICI therapy, we feel it is critical to raise awareness of the risk of RN in ICI-treated cancer patients. Careful diagnostic workup that considers multiple scenarios of disease progression and metastasis with the possible treatment effects of RT and ICI therapies will help physicians implement appropriate clinical interventions and more effectively monitor patient outcomes. We believe that the concurrent use of immunotherapy and RT had contributed to the development of RN in our patient. Cancer care is complex and with advancing science, there is a growing list of treatment options for malignancies: RT, chemotherapy, and immunotherapies. Because each of these therapeutic avenues have different mechanisms of action, the possibility for unfavorable synergy is always present. The initial thought upon our patient’s presentation was worsening metastatic disease, which would have led to continued treatment with immunotherapy or discussions of other treatment options.

## Conclusions

RN is a known complication of RT, and the risk of its development can be linked to immunotherapy use, even within the context of low-dose RT to extracranial structures involving head and neck regions. Increased vigilance in recognition and prompt diagnosis of RN is needed for all patients receiving RT, even at low doses, as misdiagnosis could lead to incorrect treatment and may greatly affect patient quality of life.

## References

[1] Giglio P, Gilbert MR (2003). Cerebral radiation necrosis. Neurologist.

[2] Ruben JD, Dally M, Bailey M, Smith R, McLean CA, Fedele P (2006). Cerebral radiation necrosis: incidence, outcomes, and risk factors with emphasis on radiation parameters and chemotherapy. Int J Radiat Oncol Biol Phys.

[3] Miyatake S, Nonoguchi N, Furuse M, Yoritsune E, Miyata T, Kawabata S, Kuroiwa T (2015). Pathophysiology, diagnosis, and treatment of radiation necrosis in the brain. Neurol Med Chir (Tokyo).

[4] Yoritsune E, Furuse M, Kuwabara H (2014). Inflammation as well as angiogenesis may participate in the pathophysiology of brain radiation necrosis. J Radiat Res.

[5] Rodríguez-Ruiz ME, Vanpouille-Box C, Melero I, Formenti SC, Demaria S (2018). Immunological mechanisms responsible for radiation-induced abscopal effect. Trends Immunol.

[6] Zhang B, Bowerman NA, Salama JK (2007). Induced sensitization of tumor stroma leads to eradication of established cancer by T cells. J Exp Med.

[7] Mothersill C, Seymour C (2001). Radiation-induced bystander effects: past history and future directions. Radiat Res.

[8] Postow MA, Callahan MK, Barker CA (2012). Immunologic correlates of the abscopal effect in a patient with melanoma. N Engl J Med.

[9] Colaco RJ, Martin P, Kluger HM, Yu JB, Chiang VL (2016). Does immunotherapy increase the rate of radiation necrosis after radiosurgical treatment of brain metastases?. J Neurosurg.

[10] Diao K, Bian SX, Routman DM (2018). Stereotactic radiosurgery and ipilimumab for patients with melanoma brain metastases: clinical outcomes and toxicity. J Neurooncol.

[11] Hodi FS, O'Day SJ, McDermott DF (2010). Improved survival with ipilimumab in patients with metastatic melanoma. N Engl J Med.

[12] Diao K, Bian SX, Routman DM (2018). Combination ipilimumab and radiosurgery for brain metastases: tumor, edema, and adverse radiation effects. J Neurosurg.

[13] Na A, Haghigi N, Drummond KJ (2014). Cerebral radiation necrosis. Asia Pac J Clin Oncol.

[14] Lubelski D, Abdullah KG, Weil RJ, Marko NF (2013). Bevacizumab for radiation necrosis following treatment of high grade glioma: a systematic review of the literature. J Neurooncol.

[15] Mou YG, Sai K, Wang ZN (2011). Surgical management of radiation-induced temporal lobe necrosis in patients with nasopharyngeal carcinoma: report of 14 cases. Head Neck.

